# Quality improvement project: Reducing non-conformities of the samples for haemostasis testing in a secondary healthcare centre through the nurses’ education in phlebotomy

**DOI:** 10.11613/BM.2020.020708

**Published:** 2020-06-15

**Authors:** Patricija Banković Radovanović

**Affiliations:** Department of transfusion medicine, General hospital Pula, Pula, Croatia

**Keywords:** quality improvement, phlebotomy, education, pre-analytical phase

## Abstract

**Introduction:**

Poor compliance to the current guidelines and lack of knowledge among nurses about proper blood sampling is set as the study hypothesis. Here is presented a quality improvement project with following aims: a) to identify the most prevalent non-conformity of the samples for haemostasis testing, b) to identify the cause of sample non-conformity, c) to perform corrective action(s) and d) to assess the effectiveness of the corrective action(s).

**Materials and methods:**

The rate of non-conformity of samples collected for haemostasis tests was established for hospital wards with inpatients. Phlebotomy practice was audited throughout anonymous questionnaire among hospital’s nurses who perform phlebotomy. Education about blood sampling was performed as a 1-hour lecture in different small groups each working day within one month. Education effectiveness was assessed through the evaluation of sample quality and is considered effective if more than half of the hospital wards significantly reduced their sample non-conformities rate.

**Results:**

Clotted sample constituted 84% of sample non-conformities. The questionnaire revealed nurses’ poor knowledge in phlebotomy. There was no difference in nurses’ knowledge regarding the level of education or work experience. Reduction in sample non-conformities was observed in 7 out of 9 wards 4 months after education; this improvement was statistically significant for 5 wards.

**Conclusion:**

Clotted sample as the most prevalent non-conformity of the samples for haemostasis testing is caused by the lack of knowledge of the nurses in several parts of the phlebotomy process. Specific education of the motivated personnel in small groups was successful and long-term effective.

## Introduction

In the past decades, lowering error frequency in laboratory medicine to approximately 0.3% is evidenced (*1*). This fact as well as wide use of the modern analysers which provide highly accurate test results led to the belief, at least as laboratory results concern, that patient safety is assured to the highest level (*2*). However, one part of the total testing process, the pre-analytical phase, is still the major source of laboratory errors which possibly lead to several unwanted clinical outcomes, consequently compromising patients’ safety (*3*). This opened the necessity to set the primary goal of laboratory specialists: achieving a high degree of uncompromised quality (*4*). Since standards provide information on what should be done, but no how to do it, various professional associations issued guidelines for several aspects of the laboratory total testing process which provide recommendations for specific performance (*5*, *6*). Such guidelines are issued *e.g.* by the Clinical and Laboratory Standards Institute (CLSI) as well as national associations (*7*-*9*). Unfortunately, laboratory praxis shows that lack of compliance to these guidelines still occurs especially in the pre-analytical phase, specifically during phlebotomy (*10*, *11*). As the most frequently occurring pre-analytical errors, sample non-conformities are recognized, and among them haemolytic samples, insufficient or inappropriate sample volume, wrong container use and undue clotting (*12*). Such sample non-conformities have a great impact on the patients’ test results which is especially critical for samples for haemostasis testing since lots of actions taken during phlebotomy may impair the reliability of these results making them false prolonged or shortened thus false normal or false pathologic (*13*). In most cases such sample non-conformities are a common cause of specimen rejection and repeatedly performed blood sampling which, besides the patient discomfort, can cause blood loss, especially in intensive care patients, possibly altering their health state (*14*). Unfortunately, this will also cause turnaround time prolongation and consequently delay in diagnosis and treatment of critical patients which in this vulnerable population could cause further aggravation contributing to the increase of the total care expenses (*15*, *16*). Thus, anticipating and reducing errors should be the continuous task of laboratory specialists. This could be achieved through the reliable error-management strategy as well as the total quality system which would effectively prevent, detect and recover errors aiming to the quality target in the pre-analytical phase like those already achieved for the analytical phase (*17*, *18*). As part of this total quality system and management, with respect to the error prevention as well as the error correction, appropriate training and education should be developed with active and corporative involvement of all participants in this process, enabling better understanding of their roles importance for the patient safety (*2*, *11*).

In this study poor compliance to the current guidelines and lack of knowledge among nurses about proper blood sampling is set as the study hypothesis.

Here is presented a quality improvement project with following aims: a) to identify the most prevalent non-conformity of the samples for haemostasis testing, b) to identify the cause of sample non-conformity, c) to perform corrective action(s) and d) to assess the effectiveness of the corrective action(s).

## Materials and methods

This study was performed in a secondary healthcare centre as a quality improvement project with initiative for the sample non-conformities identification as well as their correction and an attempt to mitigate the possibility of their repeated occurrence.

General hospital Pula consists of 14 main wards: internal medicine, surgery, anaesthesiology and intensive care, gynaecology, paediatrics, neurology, ophthalmology, otorhinolaryngology, psychiatry, infectology, transfusiology, emergency room, pathology with cytology and laboratory diagnostics. At the time of the project conduction, 527 nurses with secondary and tertiary level of education were employed, 78% and 22%, respectively. Only wards with inpatients as well as nurses whose task is to perform phlebotomy were planned to be included in this project. Wards which fulfilled the inclusion criteria and entered the study but did not order haemostasis testing in either of the three assessment periods were excluded from the study analyses.

This project was conducted in several phases. The first phase lasted from July to August 2018, when the sample conformity data was collected. The total number of samples collected for haemostasis tests, number of sample non-conformities as well as type of non-conformity were noted for each hospital ward. In the second phase which was conducted in December 2018, phlebotomy practice was audited through an anonymous questionnaire among the hospital’s nurses who perform phlebotomy. Auditing was organized in a specified hospital location were above mentioned personnel accessed to fill out the questionnaire in paper form which consisted of 10 questions about knowledge of proper phlebotomy performance (Table 1). The knowledge is considered satisfactory if at least 80% of the participants give the right answer for each subject in the questionnaire. In the third phase, education was performed by a laboratory specialist as a 1-hour lecture in small groups (20-30 participants) each working day during March 2019 in a specified hospital location. Each day of education, according to the scheduled plan, different group of nurses attended the lecture with emphasis on samples for haemostasis testing. During the lecture, participants were introduced to the first and second phase results, then they followed the presentation about proper phlebotomy performance according to the national guidelines (*9*). The participants also got acquainted with the most frequent errors during phlebotomy as well as their consequences on the quality of the sample for haemostasis tests. Additional view of the issue with sample collection through vascular collecting devices was explained theoretically and with real life examples. Lots of examples of improperly collected samples were presented (haemolysis, undue clotting, underfilling, improper labelling) as well as take-home messages which were also distributed to the hospital wards in the form of the leaflets. Time for open questions and discussion was provided as well. At the end of each lecture participants filled out an anonymous questionnaire about their satisfaction by giving grades from 1 to 5 for each of the eight aspects of education (content, performance, comprehension, usefulness, fulfilment of expectations, atmosphere, support to continue with education program and interest for new education). More than 80% of grades 4 and 5 was considered good satisfaction.

**Table 1:** The questionnaire about nurses’ knowledge of phlebotomy

**Question**
1. I consider my knowledge of phlebotomy as good yes no I am not sure
2. Fasting is necessary for the following tests: Blood type Blood count Coagulation Everything is true **Nothing is true**
3. If blood must be collected in several tubes, the order is: I first take the biggest tubes, to assure enough blood for majority of the tests I first take tubes for the tests most relevant to the patient The order of draw is not important whatsoever **Coagulation tube is always the first** The decision depends on the status of the patient’s veins
4. I perform tube labelling: **In front of the patient just before collecting blood** **In front of the patient immediately after collecting blood** I prepare tubes in advance and then I approach to the patient
5. I perform patient identification: **By asking their name** By asking if their name is “XY“ I do not need to ask the patient, I have the hospital list I read the first and last name on the bed list
6. I remove tourniquet: **Immediately after the vein puncture** When a half of the scheduled tubes are filled After finishing blood collection
7. When difficulties with the blood outflow occur: I clench the tourniquet harder to obtain better pressure I choose a new puncture spot on the same arm **I choose a new puncture spot on another arm** I fill the tubes from any vein possible until the end **I give up from blood collecting**
8. In case of the puncture problem due to the poor vein: I clench harder the tourniquet to obtain better visibility of the vein I continue spiking with the needle along the vein until I get lucky **I search for an appropriate vein on another arm** **I ask my colleague for help**
9. After the puncture spot was disinfected: I touch the vein again and introduce needle into the vein immediately after I wait for alcohol to evaporate and then touch the vein again before the puncture **I wait for alcohol to evaporate before the puncture**
10. After blood collection I mix the tube: Immediately after, 5-10 times Immediately after, 2-3 times **Mixing cycle depends on the tube type** I mix later when the blood cools Mixing is unnecessary, blood has been mixed during collection
Correct answers are bolded. There is more than one correct answer for some of the questions. Choosing one or both correct answers is considered as the affirmation of knowledge.

Education effectiveness was assessed through the evaluation of the sample quality conducted in the fourth phase during April and May, as well as from August until November 2019. Education is considered effective if more than half of the hospital wards significantly reduced their sample non-conformities rate.

Each phase was performed after meeting with wards’ head nurses as well as the hospital head nurse where results from the previous phase were explained along with the details of the next phase. Head nurses presented this information to their ward nurses. The hospital’s Ethical committee approved this study.

### Statistical analysis

Contribution of different types of the sample non-conformities was calculated as their percentage related to the total number of the sample non-conformities.

Prevalence of the sample non-conformity among the hospital wards was calculated as ratio (rate) of sample non-conformities for each hospital wards regarding their own total number of the samples collected for haemostasis testing. This was performed before and after education (immediately after as well as four months later) and the education effectiveness was calculated by using Comparison of two rates.

Knowledge of different topics within phlebotomy procedure is calculated and expressed as percentage of the participants who gave right answers in questionnaire. Differences in knowledge regarding the level of education and working experience were tested with Chi-square test, respectively. Differences proven by Chi-square test were further evaluated with Comparison of proportions.

Satisfaction of participants with the education is calculated for each assessed subject as a percentage of each grade (*1*-*5*) related to the total number of the participants.

Demographic data of the participants who approached to the questionnaire and education are presented as a percentage.

The level of significance for all calculations was defined as a P-value less than 0.05. For multiple comparison Bonferroni correction was applied and P-value less than 0.006 was set as the level of significance.

The statistical analyses were performed by using MedCalc 12.7.0.0 statistical software (MedCalc Software, Mariakerke, Belgium).

## Results

Out of 11 wards which were assessed in the first phase, 2 were excluded from the calculations due to the lack of ordering haemostasis tests in the second and third assessment period.

In the first phase 418 out of 2226 samples for haemostasis testing did not conform to the quality standards. Among all observed non-conformities, clotted sample is the most contributing one (84%), followed by haemolysis (14%) and undefiled tube (2%).

**Figure 1:**
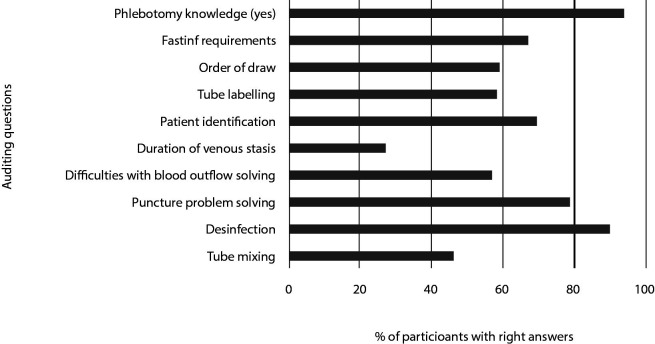
Results of the questionnaire among nurses (N = 147) about their knowledge of phlebotomy. First upper horizontal band represents nurses’ self-declared perception of knowledge. Other nine bands correspond to the real knowledge in different parts of the phlebotomy procedure. Vertical line indicates desired percentage of participants with affirmed knowledge.

To the first questionnaire (Table 1) 147 out of 431 nurses approached. Their demographic data are presented in the Table 2. Although 94% nurses claimed that they have good knowledge in phlebotomy, the cut-off of 80% of the participants with the correct answers satisfied only for the question No.9 (90%). Question No.6 was the one with the lowest percentage of the correct answers (27%). All results of the questionnaire are presented in the Figure 1. Difference in knowledge regarding level of education was found only for question No.9. (P = 0.023). Further evaluation revealed that nurses with secondary level of education showed 13% higher knowledge regarding disinfection of the puncture spot compared to the nurses with tertiary level of education (95% CI = 2.2-23; P = 0.030). No significant difference was evidenced regarding the work experience (P-value for questions 1 to 10 was ranging from 0.073 to 0.743).

**Table 2:** Demographic data of the participants in each phase of the quality improvement project

**Participants in the knowledge questionnaire** **(N = 147)**	**%**
Level of education	
Secondary	64
Tertiary	36
Work experience (years)	
< 1	2.7
1-5	17
6-10	12
11-15	13
16-20	7.3
21-25	13
26-30	12
31-35	10
36-41	13
**Participants in education (N = 368)**	
Gender (F)	89
Level of education	
Secondary	77
Tertiary	23
**Participants in the questionnaire of satisfaction (N=328)**	**/**
N - number of participants in each phase and is a base for percentage calculation. F - female.	

The education was conducted with 368 out of 431 nurses whose demographic data are presented in the Table 2. In the sample quality assessment immediately after education 478 out of 2632 samples for haemostasis testing did not conform to the quality standards, yielding the difference of 1% less sample non-conformities comparing to the period before the education (P = 0.391). Table 3 shows that 4 out of 9 wards lowered the ratio of the sample non-conformity, with the statistical significance only for two of them (columns 0-1^†^ and related P-value). Four months after the education, 485 sample non-conformities out of 4241 samples for haemostasis testing were revealed, yielding the difference of 8% less sample non-conformities comparing to the period before education (P < 0.001). As the Table 3 shows, 7 out of 9 wards made improvements in their phlebotomy performance, which was statistically significant for 5 wards (columns 0-2^††^ and related P-value) with higher frequency of intensive care patients. Wards with mainly routine patients did not show statistically significant improvement.

**Table 3:** Effectiveness of the education assessed by evaluation of the difference in the sample non-conformity

**HW**	**NC/TS ratio**			**Difference in sample nonconformity^§^**			
**Before education (0)**	After education (1)*	After education (2)**	**0-1^†^**	**P**	**0-2^††^**	**P**
**1**	0.1026	0.1428	0.0737	- 0.0402	0.022	0.0289	0.026
**2**	0.3598	0.2346	0.1551	0.1252	< 0.001	0.2047	< 0.001
**3**	0.1550	0.1407	0.0792	0.0143	0.591	0.0758	< 0.001
**4**	0.2154	0.0419	0.0279	0.1735	< 0.001	0.1875	< 0.001
**5**	0.2107	0.2227	0.2115	- 0.0120	0.780	- 0.0008	0.982
**6**	0.1912	0.2500	0.1339	- 0.0588	0.446	0.0573	0.319
**7**	0.2759	0.3654	0.1413	- 0.0895	0.503	0.1346	0.129
**8**	0.2947	0.1987	0.1770	0.0960	0.125	0.1177	0.044
**9**	0.1304	0.4615	0.1500	- 0.3311	0.056	- 0.0196	0.864
HW - hospital ward. NC - number of samples for haemostasis testing non-conform to the quality standards. TS - total number of the samples for haemostasis testing. *sample quality was assessed immediately after education. **sample quality was assessed four months after education. ^†^difference between NC/TS ratios before and immediately after education. ^††^difference between NC/TS ratios before and four months after education. ^§^Comparison of two rates has been used as the test. P < 0.05 was considered statistically significant.							

Out of 368 participants, 328 fulfilled questionnaires about their satisfaction with the education and these results are presented in the Table 4.

**Table 4:** Participants’ satisfaction with the education (N = 328)

**Assessment**	**Satisfaction (N = 328)**				
**subject**	**Grade 1 (%)**	**Grade 2 (%)**	**Grade 3 (%)**	**Grade 4 (%)**	**Grade 5 (%)**
Content	0	0.3	3.4	9.1	87
Performance	0	0.3	1.8	7.6	90
Comprehension	0	0	0.6	5.8	94
Usefulness	0.3	0.6	4.0	9.5	86
Fulfilment of expectations	0	1.2	4.9	12	82
Atmosphere	0.3	1.5	4.0	11	83
Support to continue with education program	0	0.6	4.0	9.1	86
Interest for new education	0.9	0.6	5.8	10	83

## Discussion

This study identified clotted sample as the most prevalent non-conformity of the samples for haemostasis testing due to the poor performance of several parts in the phlebotomy process which was revealed throughout the questionnaire. In fact, the initial questionnaire showed that nurses are poorly informed with the duration of venous stasis, order of draw, tube mixing and the procedure when difficulties with blood flow occur. Each of these factors contributes to the clotting of the sample for haemostasis testing unless performed properly (*2*, *18*). These are also parts of the questionnaire with the lowest demonstrated level of knowledge and hence can be assumed as the cause of the most prevalent sample non-conformity in this study. Although approximately one third of the nurses who perform phlebotomy took the questionnaire, the results from the questionnaire were compatible with the first sample quality assessment, thus reflecting and explaining the existing problem. Nevertheless, the fact that the ratio of the participating nurses regarding their level of education slightly differed from their ratio in the whole nurses’ population it could be considered as limitation. From one perspective this could have effect on the representativeness of the population, however, the questionnaire resulted with no difference in knowledge in phlebotomy regarding the level of education and work experience. This opens the other perspective in which the conclusion about the evidenced representativeness of the audited group becomes more plausible.

Since the level of knowledge did not meet criteria which were set as satisfactory, it was more than obvious that education is needed. Specific education for the phlebotomy errors correction resulted with the reduction in the sample non-conformity occurrence. The assessment of the education effectiveness immediately after education showed minor improvement, since only two hospital wards significantly reduced sample non-conformity. But this was expected since the evaluation followed shortly after education even though it lasted for a certain time. As opposite, the second cycle of the education effectiveness assessment four months after education showed higher reduction of sample non-conformity which was significant for more than a half hospital wards. These results met the criteria set for the education effectiveness and, what is probably more important, the improvement was sustained four months after the education and even more since the assessment lasted for further four months. This is very satisfactory and promising, since the education was performed as a 1-hour presentation per participant. It seems that some additional factors contributed to the successful reduction of the pre-analytical errors. The education within small groups has advantages since it allows better communication and knowledge transference. Furthermore, the program can be tailored specifically for the topics of interest. One other thing that should not be neglected is interest and susceptibility of the education participants. It was the exit questionnaire that showed that participants were highly satisfied with the current education and that they are interested in the new education which reflects their professionality and willingness to improve their performance. The participants’ interest and motivation, which was obtained through the continuous information throughput over the whole project, were probably the key factors for the long-term education effectiveness which was proven with the ultimate results. Moreover, the specific approach to the participants and active inclusion of the hospital superiors in the whole process also represents the added value. A well-informed person has a better probability to understand their role and embrace the change, to gain personal satisfaction and to feel respected; all of this belongs to the Change management principles and will lead to success (*19*).

Several studies showed that education on venous blood specimen collection gained significant improvements in this practice (*20*-*25*). In these studies, education was performed as a 1- or 2-hours lecture, except for the study of Lima-Oliveira *et al* whose education was consisted of an 8-hour personal training, and Bostic *et al* who gave the nurses leaflets with information on how to solve specific error. All these studies reported improvement after education which corroborate findings of this study that education does not have to be extensive to be successful. However, these studies differ in the education effectiveness assessment. Lima-Oliveira *et al* assessed the education effectiveness by the observation of the nurses which is a good way to see what a person does in each phase, but it is more appropriate to assess the person’s performance prior to the education (*21*). Since the person is aware of being observed, he/she could do everything properly, but this cannot assure that a person will sustain the good performance when nobody observes. Arslan *et al* observed pre-analytical error rates before and one month after the training which could reflect only short-term effect (*22*). In their study, Bölenius *et al* included a face-to-face interview which gained more information about achieved knowledge, but it did not give information on the reduction in the error occurrence hence it does not properly assess education effectiveness (*26*). In this study the questionnaire was used only to assess the participants’ performance before education as well as their satisfaction with the education. The education effectiveness was assessed more objectively aiming to obtain the data about long-term effect.

In other study, Bölenius *et al* have measured a minor effect of the education on the sample quality and Abbas *et al.* measured no effect at all. In these studies, however, pre-analytical errors were at a low level even before the education and a significant improvement is not expected as Lilo et al also concluded in their study (*27*, *28*).

Regarding that, although improvement after education was satisfactory in this study, there is still enough space for further improvements. This is in concordance with the recommendations of the working group for the pre-analytical phase of the European Federation of Clinical Chemistry and Laboratory Medicine (EFLM) which believes that “there is a need to assess the quality of current practices of phlebotomy across Europe as well as engagement of the national EFLM societies in continuous education of healthcare phlebotomy staff.” (*29*).

The cost-benefit of obtained improvement which could provide additional value to these results is not evaluated here which is considered as limitation of this study.

This study gained several findings. Clotted sample is the most prevalent non-conformity of the samples for haemostasis testing. This non-conformity is caused by the lack of knowledge of the nurses in parts of the phlebotomy process which are critical to avoid the sample clotting. Specific education of the motivated personnel in small groups which is performed as the corrective action has shown to be successful and long-term effective.
